# Genome-wide expression profiling in colorectal cancer focusing on lncRNAs in the adenoma-carcinoma transition

**DOI:** 10.1186/s12885-019-6180-5

**Published:** 2019-11-06

**Authors:** Alexandra Kalmár, Zsófia Brigitta Nagy, Orsolya Galamb, István Csabai, András Bodor, Barnabás Wichmann, Gábor Valcz, Barbara Kinga Barták, Zsolt Tulassay, Peter Igaz, Béla Molnár

**Affiliations:** 1 2nd Department of Internal Medicine, Semmelweis University, Szentkirályi str. 46, Budapest, 1088 Hungary; 2 Molecular Medicine Research Unit, Hungarian Academy of Sciences, Budapest, Hungary; 3 Department of Physics of Complex Systems, Eötvös Loránd University, Budapest, Hungary

**Keywords:** Long non-coding RNA, lncRNA, Colorectal cancer, Colorectal adenoma, Human transcriptome Array 2.0, Microarray, qRT-PCR, UCA1, Immunohistochemistry, *In situ* hybridization

## Abstract

**Background:**

Long non-coding RNAs (lncRNAs) play a fundamental role in colorectal cancer (CRC) development, however, lncRNA expression profiles in CRC and its precancerous stages remain to be explored. We aimed to study whole genomic lncRNA expression patterns in colorectal adenoma–carcinoma transition and to analyze the underlying functional interactions of aberrantly expressed lncRNAs.

**Methods:**

LncRNA expression levels of colonic biopsy samples (20 CRCs, 20 adenomas (Ad), 20 healthy controls (N)) were analyzed with Human Transcriptome Array (HTA) 2.0. Expression of a subset of candidates was verified by qRT-PCR and *in situ* hybridization (ISH) analyses. Furthermore, *in silico* validation was performed on an independent HTA 2.0, on HGU133Plus 2.0 array data and on the TCGA COAD dataset. MiRNA targets of lncRNAs were predicted with miRCODE and lncBase v2 algorithms and miRNA expression was analyzed on miRNA3.0 Array data. MiRNA-mRNA target prediction was performed using miRWALK and c-Met protein levels were analyzed by immunohistochemistry. Comprehensive lncRNA-mRNA-miRNA co-expression pattern analysis was also performed.

**Results:**

Based on our HTA results, a subset of literature-based CRC-associated lncRNAs showed remarkable expression changes already in precancerous colonic lesions. In both Ad vs. normal and CRC vs. normal comparisons 16 lncRNAs, including downregulated LINC02023, MEG8, AC092834.1, and upregulated CCAT1, CASC19 were identified showing differential expression during early carcinogenesis that persisted until CRC formation (FDR-adjusted *p* < 0.05). The intersection of CRC vs. N and CRC vs. Ad comparisons defines lncRNAs characteristic of malignancy in colonic tumors, where significant downregulation of LINC01752 and overexpression of UCA1 and PCAT1 were found. Two candidates with the greatest increase in expression in the adenoma-carcinoma transition were further confirmed by qRT-PCR (UCA1, CCAT1) and by ISH (UCA1). In line with aberrant expression of certain lncRNAs in tumors, the expression of miRNA and mRNA targets showed systematic alterations. For example, UCA1 upregulation in CRC samples occurred in parallel with hsa-miR-1 downregulation, accompanied by c-Met target mRNA overexpression (*p* < 0.05).

**Conclusion:**

The defined lncRNA sets may have a regulatory role in the colorectal adenoma-carcinoma transition. A subset of CRC-associated lncRNAs showed significantly differential expression in precancerous samples, raising the possibility of developing adenoma-specific markers for early detection of colonic lesions.

## Background

The incidence and mortality of colorectal cancer (CRC) are continuously increasing with approximately 1.4 million new CRC cases and 700.000 registered deaths worldwide [1]. Therefore, identification of molecular markers of CRC that might enhance the objective classification or the early detection of the disease remains highly relevant, as CRC is one of the most curable cancers if detected early [2]. Besides the commonly investigated molecular markers, such as DNA mutations, DNA methylation or mRNA expression alterations, interest is growing in an emerging novel class of non-coding RNAs, long non-coding RNAs (lncRNAs) [3–5].

LncRNAs are defined as transcripts longer than 200 base pairs without an open reading frame [6]. This class of non-coding RNAs represents a diverse group with known and predicted functions for gene expression regulation [7–9]. According to experimental data, lncRNAs can interact with DNA, RNA and also with proteins and can either promote or inhibit transcription [10]. In contrast to miRNA-mediated regulation, the function and mechanism of action of certain lncRNAs can be diverse; lncRNAs are involved in genomic imprinting, transcriptional regulation, protein scaffolding, maintenance of hetero-euchromatin balance, can function as a miRNA sponge, and also mediate disease-derived alterations of mRNAs, miRNAs and proteins [9, 11]. Dysregulated lncRNAs are known to contribute to CRC formation through the disruption of various signaling cascades including Wnt/β-catenin, EGFR/IGF-IR (KRAS and PI3K pathways), TGF-β, p53 and Akt signaling pathways, and also via influencing the epithelial-mesenchymal transition program [12]. To date, 172.216 human lncRNA transcripts have been identified according to NONCODEv5 database [13] and their number continues to increase. Recent studies have demonstrated that several lncRNAs have a key regulatory role in various diseases including CRC [14]. During the carcinogenesis, lncRNA expression alterations affect major biological processes, and therefore. lncRNAs are considered as powerful molecular markers and also potential therapeutic targets in various cancers [3, 15].

In the present study, we aimed to determine the differentially expressed lncRNAs at the whole genome level focusing on the colorectal adenoma-carcinoma transition to identify lncRNAs showing specific alterations only in CRC tissue and common lncRNA patterns characteristic both in benign and malignant colonic neoplasms. Furthermore, we validated the lncRNA expression alterations by qRT-PCR, *in situ* hybridization, on an independent HTA 2.0 dataset, HGU133 Plus2.0, and The Cancer Genome Atlas (TCGA) Colon adenocarcinoma (COAD) datasets. We also report an association between the dysregulated lncRNAs and mRNA, miRNA and protein expression.

## Methods

### Sample collection

During routine screening endoscopy examinations biopsy samples were collected from patients with untreated colorectal cancer (*n* = 20; Astler-Coller modified Dukes B-D), with colorectal adenomas (*n* = 20; tubulovillous: *n* = 9, tubular: *n* = 11; with low-grade dysplasia: *n* = 18, with high-grade dysplasia: *n* = 2), and from healthy donors (*n* = 20). Healthy donors had been referred to the outpatient clinic with constipation, rectal bleeding or chronic abdominal pain. Ileocolonoscopy showed normal macroscopic appearance, and no abnormal histologic changes were detected in biopsy samples. None of the healthy patients had familial history of CRC. Biopsies were immediately put in RNALater stabilization reagent (Qiagen GmbH, Hilden, Germany) and stored at − 80 °C. Written informed consent was provided by all patients. The study was approved by the local ethics committee (Semmelweis University Regional and Institutional Committee of Science and Research Ethics; Nr.: ETT TUKEB 23970/2011). The clinicopathological data for the analyzed sample set are reported in Table 1.

**Table 1:** Clinicopathological data of patients involved in the study

Case number #	Age-range	Diagnosis	Adenoma grade /Astler-Coller modified Dukes stage
N1	24-76 years	healthy colonic tissue	
N2		healthy colonic tissue	
N3		healthy colonic tissue	
N4		healthy colonic tissue	
N5		healthy colonic tissue	
N6		healthy colonic tissue	
N7		healthy colonic tissue	
N8		healthy colonic tissue	
N9		healthy colonic tissue	
N10		healthy colonic tissue	
N11		healthy colonic tissue	
N12		healthy colonic tissue	
N13		healthy colonic tissue	
N14		healthy colonic tissue	
N15		healthy colonic tissue	
N16		healthy colonic tissue	
N17		healthy colonic tissue	
N18		healthy colonic tissue	
N19		healthy colonic tissue	
N20		healthy colonic tissue	
Ad1	42-88 years	Tubular adenoma	low-grade
Ad2		Tubulovillous adenoma	high-grade
Ad3		Tubulovillous adenoma	low-grade
Ad4		Tubulovillous adenoma	low-grade
Ad5		Tubulovillous adenoma	high-grade
Ad6		Tubular adenoma	low-grade
Ad7		Tubulovillous adenoma	low-grade
Ad8		Villous / tubulovillous adenoma	low-grade
Ad9		Tubulovillous adenoma	low-grade
Ad10		Tubular adenoma	low-grade
Ad11		Tubular adenoma	low-grade
Ad12		Tubular adenoma	low-grade
Ad13		Tubular adenoma	low-grade
Ad14		Tubular adenoma	low-grade
Ad15		Tubular adenoma	low-grade
Ad16		Tubular adenoma	low-grade
Ad17		Tubular adenoma	low-grade
Ad18		Tubular adenoma	low-grade
Ad19		Tubulovillous adenoma	low-grade
Ad20		Tubulovillous adenoma	low-grade
T1	46-87 years	Colorectal adenocarcinoma	Dukes C2
T2		Colorectal adenocarcinoma	Dukes B2
T3		Colorectal adenocarcinoma	Dukes C
T4		Colorectal adenocarcinoma	Dukes B2
T5		Colorectal adenocarcinoma	Dukes D
T6		Colorectal adenocarcinoma	Dukes C
T7		Colorectal adenocarcinoma	Dukes B2
T8		Colorectal adenocarcinoma	Dukes D
T9		Colorectal adenocarcinoma	Dukes D
T10		Colorectal adenocarcinoma	Dukes B2
T11		Colorectal adenocarcinoma	Dukes C
T12		Colorectal adenocarcinoma	Dukes B1
T13		Colorectal adenocarcinoma	Dukes D
T14		Colorectal adenocarcinoma	Dukes D
T15		Colorectal adenocarcinoma	unknown
T16		Colorectal adenocarcinoma	Dukes B2
T17		Colorectal adenocarcinoma	Dukes B2
T18		Colorectal adenocarcinoma	Dukes B1
T19		Colorectal adenocarcinoma	Dukes B1
T20		Colorectal adenocarcinoma	Dukes C

### RNA isolation, quality and quantity analyses

Total RNA including the microRNA (miRNA) fraction was isolated with High Pure miRNA isolation kit (Cat no: 05080576001, Roche, Penzberg, Germany) using the one-column protocol according to the manufacturer’s recommendation. RNA quantity was measured on a Qubit fluorometer with the Qubit™ RNA Assay Kit (Life Technologies, Eugene, OR, USA) and also on the NanoDrop-1000 instrument (Thermo Fisher Scientific Inc., Waltham, USA) to determine the purity values (OD260/280, OD260/230). RNA quality analysis was performed on an Agilent Bioanalyzer microcapillary electrophoresis system with the RNA 6000 Pico Kit (Agilent, Santa Clara, CA, USA).

### Microarray experiment

For lncRNA expression profiling Human Transcriptome Array 2.0 (HTA 2.0) experiments were performed with 100 ng total RNA sample input according to the manufacturer’s instructions. For single-stranded complementary DNA (sscDNA) synthesis 15 μg complementary RNA (cRNA) was used and 5.5 μg fragmented and labeled sscDNA sample was hybridized to Human Transcriptome Array 2.0 microarrays (Affymetrix, Santa Clara, CA, USA) for 16 h at 45 °C with 60 rpm rotation in the Hybridization Oven (Affymetrix). Microarrays were washed and stained with GeneChip® Hybridization, Wash, and Stain Kit reagents according to the FS450_0001 protocol using the Fluidics Station 450 instrument (Affymetrix). Scanning was performed with GeneChip Scanner 3000 (Affymetrix). The dataset was uploaded to the Gene Expression Omnibus (GEO) data repository: GEO ID GSE100179. Raw CEL file normalization was performed with the Expression Console (Affymetrix, version: 1.4.1.46) with Gene Level – Default: RMA algorithm in order to generate .chp files. LncRNA expression level comparisons were made for adenoma vs. healthy, CRC vs. adenoma and CRC vs. healthy samples (FDR adjusted *p* < 0.05; log2FC ≤ − 1 or log2FC ≥ 1). LncRNA annotation and classification (with the inclusion of non-coding, 3 prime overlapping ncRNA, antisense, lincRNA, sense intronic, sense overlapping and bidirectional lncRNA subclasses of lncRNAs) were performed using the BioMart data mining tool on the basis of the current Ensembl database (Ensembl release 93 - July 2018 using GRCh38.p12 human genome version) and was further confirmed with the Netaffx database.

### qRT-PCR validation

Certain lncRNAs with significant expression alterations in the three comparisons were further studied by qRT-PCR. 500 ng total RNA was reverse transcribed with TaqMan MicroRNA Reverse Transcription kit (Applied Biosystems, Foster City, CA, USA). Absolute quantification was performed using LightCycler 480 Probes Mastermix (2x), Resolight dye (40x), and primers (200 nM final concentration; Table 2.) with 5 ng cDNA/reaction with the following thermal cycling conditions: enzyme activation: 95 °C for 10 min, 45 cycles of amplification: 95 °C for 10 s, T_annealing_ (CCAT1: 57 °C, LINC00261: 59 °C, UCA1: 61 °C) for 30 s and signal detection at 72 °C for 1 s and cooling at 40 °C for 30 s. As the normalization of expression during lncRNA quantification is critical, absolute quantification was performed. After PCR amplification of standard samples, amplicons were analyzed by 2% agarose gel electrophoresis and were purified with Agencourt AmpureXP Beads (Beckman Coulter, Brea, USA) according to the standard PCR purification protocol. The expression levels of lncRNAs in the analyzed samples were quantified after establishing standard curves with serial dilution of standard samples (with 2, 10,^1^ 10,^2^ 10,^3^ 3 × 10,^3^ 10^4^ molecules/reaction) calculated based on Qubit fluorometry results. Finally, we validated the standard curves of each amplicon and analyzed the amplification efficiency, R,^2^ and the slope. LightCycler 480 absolute quantification software was used to calculate copy numbers in the analyzed samples.

**Table 2:** Primer sequences of qRT-PCR validation

	Forward primer	Reverse primer
CCAT1	TCACTGACAACATCGACTTTGAAG	GGAGAAAACGCTTAGCCATACAG
UCA1	AATGCACCCTAGACCCGAAA	TCACAGGGGTTACAATGGCT
LINC00261	AATAAATGCGGGGATGCCTC	CTGGGAAGCCTAGGTCTGTT

### *In situ* hybridization with automated RNAscope and immunofluorescence

The *in situ* hybridization (ISH) analyses were performed in collaboration with Boye Schnack Nielsen, Bioneer A/S, Hørsholm, Denmark. Five μm thick FFPE sections were processed for RNAscope ISH in a Ventana Discovery Ultra instrument (Roche, Basel, Switzerland) [16]. The following RNAscope probes were obtained from ACD, Biotechne (Newark, CA, USA): UCA1 (Urothelial cancer associated 1, NR_015379.3, target region: 659 − 2289, 20 zz pairs), dapB (a Bacillus subtilis gene, 414 – 862, 10 zz pairs), and PPIB (Cyclophilin B, 139 – 989, 16 zz pairs), and incubated on tissue sections as recommended by the manufacturer. The RNAscope probes were detected using the HRP kit and Discovery-rhodamine substrate (Roche). For cytokeratin immunofluorescence, the AE1/3 mouse monoclonal antibody (Dako-Agilent, Glostrup, Denmark) was used at 1:200 and detected with Alexa-488 conjugated anti-mouse Ig (Jackson Immunoresearch, West Grove, PA). The stained sections were mounted with a DAPI-containing anti-fade solution, ProLong Gold (Thermo Fisher Scientific, Waltham, MA, USA). Digital whole slides were obtained with a Pannoramic Confocal (3DHISTECH Ltd., Budapest, Hungary) slide scanner using a 40x objective, and the localization of expression was examined in these, as well as representative images were acquired from these digital slides.

### *In silico* validation

#### lncRNA expression on an independent HTA 2.0 dataset

Our HTA 2.0 results were compared with the results of Condorelli et al. from GEO database (GSE73360) [17] of 37 CRC biopsies from 27 patients, and 19 adjacent normal mucosa biopsies (at distance of 3-6 cm from the tumor) that were collected directly after surgical resection. Linear correlation was analyzed between the two datasets.

#### lncRNA expression on HGU133Plus 2.0 dataset

For * in silico* validation, expression levels of lncRNAs were also analyzed on GSE37364 HGU133Plus 2.0 microarray (Affymetrix) dataset of 65 human colonic biopsy samples (27 CRCs and 38 normal donors without evidence of disease) [18]. Alignment of the probesets of the different platforms was performed using the BioMart data mining tool based on the current Ensembl database (Ensembl release 93 - July 2018 using GRCh38.p12 human genome version). Among the significant lncRNA expression alterations identified on HTA 2.0 arrays, 11 associated probesets could be found on the HGU133Plus2.0 arrays representing 10 lncRNAs. Linear correlation between the two microarray platforms was also analyzed.

#### lncRNA expression on TCGA dataset

Selected lncRNAs showing altered expression on HTA 2.0 arrays were analyzed *in silico *using The Cancer Genome Atlas (TCGA) colon & rectum adenocarcinoma gene expression RNAseq dataset. We have used the COAD (*n* = 463) subset of the lncRNA dataset from Yan et al. which was compiled from 5.037 human tumor specimens across 13 cancer types in TCGA [19, 20]. Out of the 37 lncRNAs identified in our cohort showing significant under- or overexpression in the CRC vs. N comparison, 16 could be detected in TCGA data. Linear correlation was analyzed between the datasets.

#### LncRNA-mRNA-miRNA co-expression analysis

The HTA 2.0 microarray provides expression data of lncRNAs and mRNAs allowing the parallel analysis of both from individual samples. In order to assess lncRNA/mRNA relationships, co-expression networks were constructed based on Pearson-correlation calculation. The top 50 negatively and top 50 positively associated, predicted mRNA targets of the significantly (*p* < 0.05) altered, qRT-PCR validated lncRNAs (UCA1 [TC19000279.hg.1, TC19002012.hg.1] and CCAT1 [TC08001627.hg.1]) were selected. These lncRNAs were visualized with the associated mRNAs by using igraph in the R environment and Gene Ontology (GO) functional analysis was performed using Netaffx database (Affymetrix). On the other hand, MIRCODE, lncBase v2 predicted and lncBase v2 experimental algorithms were used to predict miRNAs associated with UCA1 lncRNA. The expression of selected miRNAs was analyzed on the miRNA 3.0 microarray (GSE83924) dataset from Nagy et al. [21]. The miRWALK database was used for miRNA-mRNA target prediction involving 12 existing miRNA-target prediction algorithms (DIANA-microTv4.0, DIANA-microT-CDS, miRanda-rel2010, mirBridge, miRDB4.0, miRmap, miRNAMap, doRiNA, PicTar2, PITA, RNA22v2, RNAhybrid2.1 and Targetscan6.2) and containing experimentally verified data from existing resources (miRTarBase, PhenomiR, miR2Disease and HMDD) [22].

### Immunohistochemistry

6 μm thick slides cut from tissue microarray (TMA) blocks of normal (*n* = 20), adenoma (*n* = 20) and CRC (*n* = 20) patients were deparaffinized and rehydrated. Antigen retrieval was performed in TRIS-EDTA buffer (pH 9.0) using a microwave oven (900 W for 10 min, 340 W for 40 min). Samples were incubated with anti-c-Met antibody (rabbit polyclonal, ab4193, Abcam, Cambridge, UK) diluted 1:800 for 60 min at 37 °C. EnVision + HRP system (Labeled Polymer Anti-Mouse, K4001, Dako) and diaminobenzidine-hydrogen peroxidase–chromogen substrate system (Cytomation Liquid DAB + Substrate Chromogen System, K3468, Dako) were applied followed by hematoxylin counterstaining. Slides were digitally scanned using the Pannoramic 250 Flash instrument (software version 1.11.25.0, 3DHISTECH Ltd., Budapest, Hungary), and analyzed with a Pannoramic Viewer (v. 1.11.43.0. 3DHISTECH Ltd.) digital microscope based on Q-score method; scored by multiplying the percentage of positive cells (P) by the intensity (I: + 3 (strong), + 2 (moderate), + 1 (weak), 0 (no staining)). Formula: Q = P x I; maximum = 300) as described earlier [23].

### Statistical analysis

For data distribution analysis the Kolmogorov-Smirnov test was applied. Due to the normal distribution, Student’s t-test was applied for the pairwise comparison with Bonferroni and Hochberg correction. FDR adjusted *p*-values lower than 0.05 were considered as significant. Pearson-correlation was calculated and lncRNA-mRNA-miRNA co-expression network was constructed by igraph package in the R environment.

## Results

### Expression of known CRC-associated lncRNAs in the colorectal adenoma and carcinoma samples

As the expression of known CRC-associated lncRNAs has not been studied yet in precancerous adenoma samples, in the present study we aimed to analyze the adenoma-specific alterations with a special focus on adenoma-carcinoma transition.

**Fig. 1:**
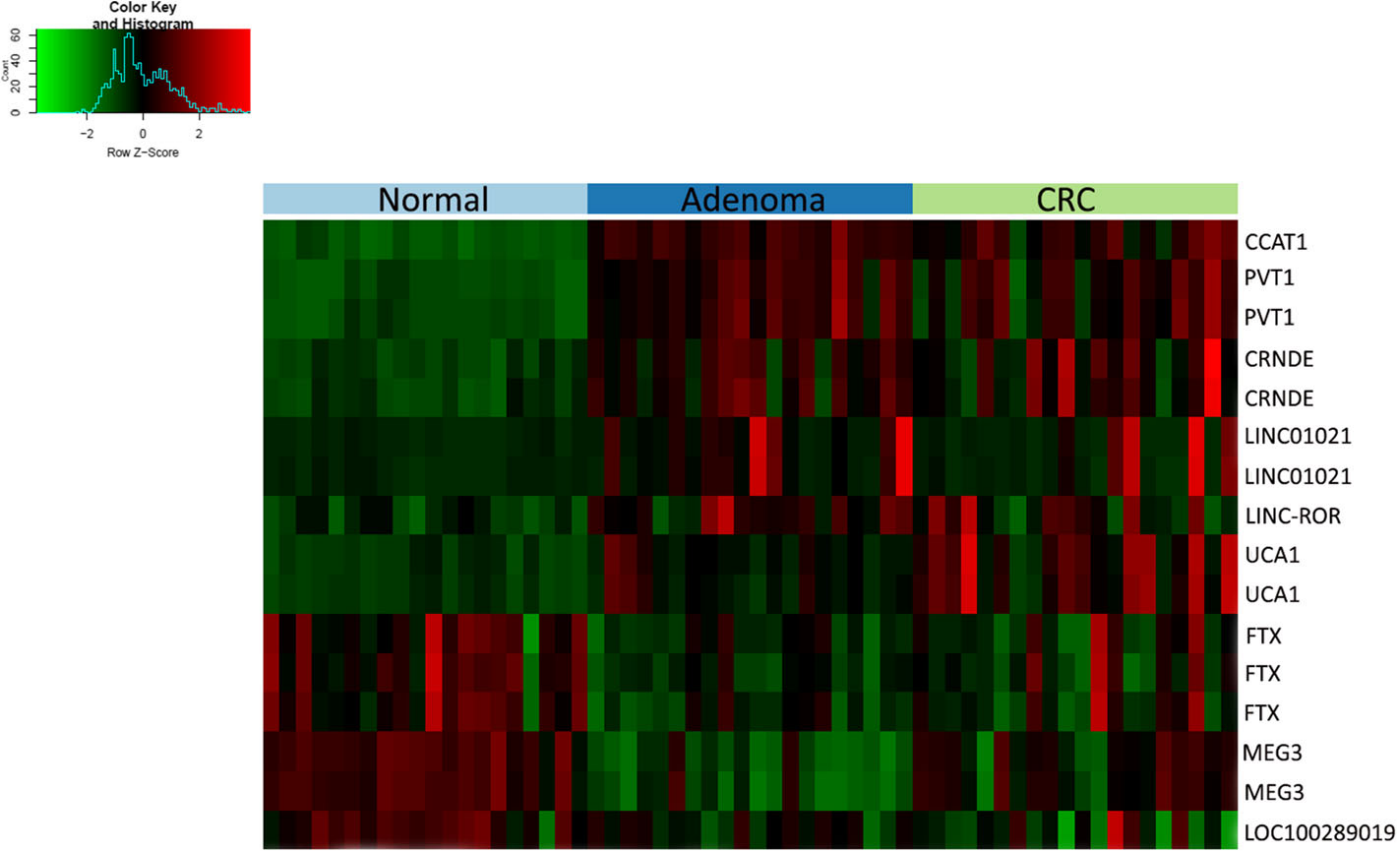
CRC-associated lncRNA expression in colorectal adenoma and in CRC samples. Intensity values on the color scale were as follows: red – high intensity, black – intermediate intensity, green – low intensity. A subset of literature-based colorectal cancer-associated lncRNAs showed significant expression difference already between normal and adenoma samples (*p* < 0.01)

Previous comprehensive studies analyzing healthy colon and CRC tissue samples revealed differentially expressed lncRNAs, so-called CRC-associated lncRNA markers [24–26]. First, the expression of these literature-based lncRNA markers was studied on adenoma and CRC samples as part of our whole transcriptome analysis. A subset of lncRNAs from our Human Transcriptome Array 2.0 study (CCAT1, PVT1, CRNDE; LINC01021, LINC-ROR, UCA1, FTX, MEG3, LOC100289019) showed remarkable expression changes already in the precancerous colonic lesions (*p* < 0.01) (Fig. 1).

### Differentially expressed lncRNAs in the colorectal adenoma-carcinoma sequence progression

**Fig. 2:**
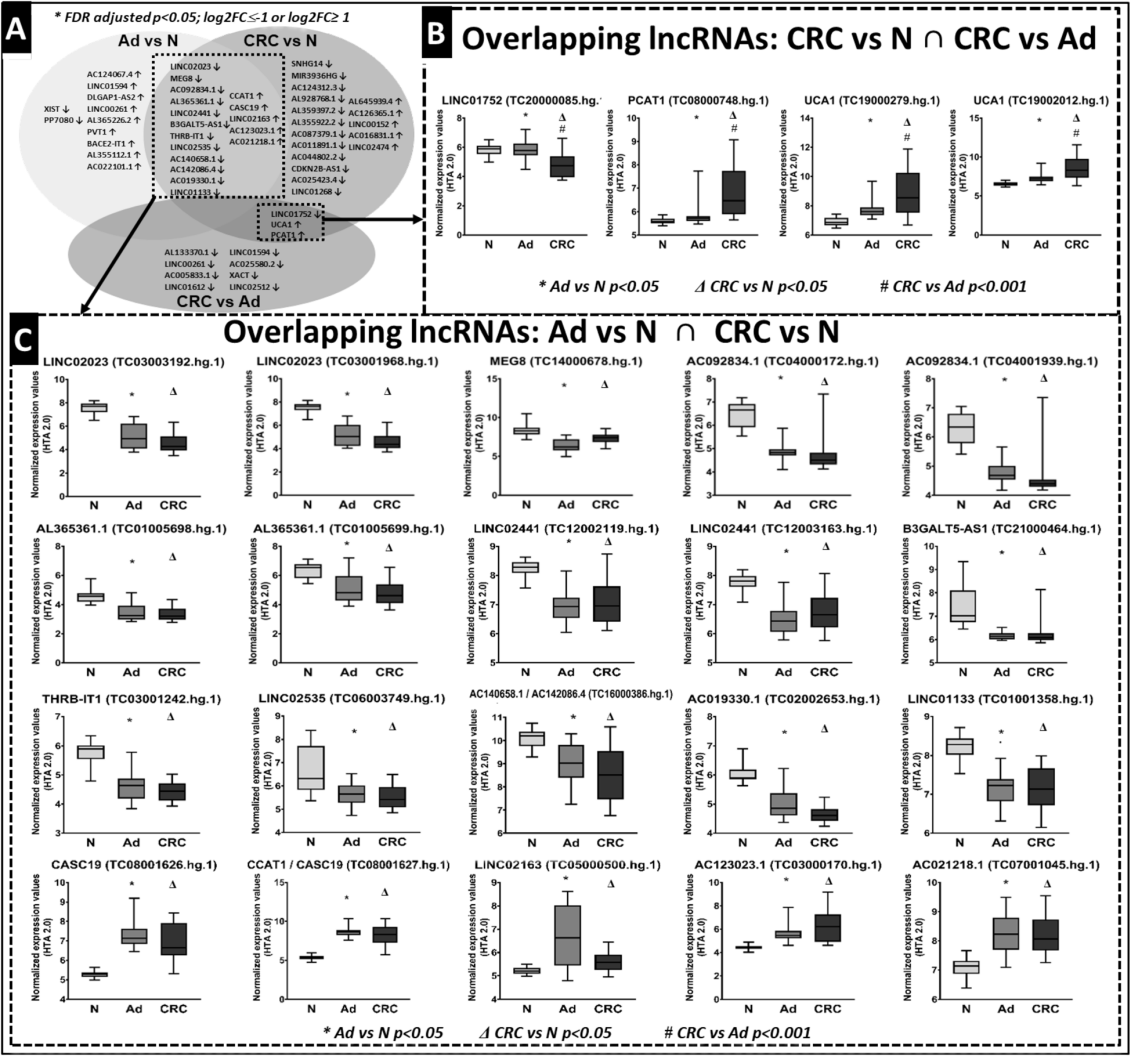
**A**) Venn-diagram of the significantly altered lncRNAs in Ad vs. N, CRC vs. N and CRC vs. Ad comparisons (FDR adjusted *p*-value< 0.05; log2FC ≤ −1 or log2FC ≥ 1). Boxplot representation of the overlapping lncRNAs showing differential expression in **B**) CRC vs. N and CRC vs. Ad and **C**) Ad vs. N and CRC vs. N comparisons

On the basis of the HTA 2.0 results, 54 lncRNAs were found to be differentially expressed along the colorectal adenoma-carcinoma sequence (FDR adjusted *p*-value< 0.05, log2FC ≥ 1 or log2FC ≤ − 1) (Additional file 1: Figure S1, Additional file 2: Table S1). In order to focus on the lncRNA marker candidates, whose dysregulation might be driver in the development of CRC, a group of 17 lncRNAs was identified as overlapping between adenoma vs. normal and CRC vs. normal comparisons showing differential expression early that persisted until CRC formation: LINC02023, MEG8, AC092834.1, AL365361.1, LINC02441, B3GALT5-AS1, THRB-IT1, LINC02535, AC140658.1, AC142086.4, AC019330.1 and LINC01133 were downregulated and CCAT1, CASC19, LINC02163, AC123023.1 and AC021218.1 were upregulated in adenoma and also in CRC samples compared to the healthy controls (FDR adjusted *p* < 0.05, log2FC ≤ − 1 or log2FC ≥ 1). Three lncRNAs (downregulated LINC01752, and overexpressed UCA1 and PCAT1) were detected in both CRC vs. N and CRC vs. Ad comparisons, suggesting that these changes might be early markers of colonic carcinogenesis (Fig. 2, Table 3).

**Table 3:** lncRNAs with potential key role in the colorectal adenoma-carcinoma transition

Overlapping lncRNAs in the CRC vs. N & Ad vs. N comparisons				
HTA 2.0 ID	Annotation	lncRNA name	log2FC (Ad vs. N)	log2FC (CRC vs. N)
TC03003192.hg.1	LINC02023	Long intergenic non-protein coding RNA 2023	-2.387**	-2.960***
TC03001968.hg.1	LINC02023	Long intergenic non-protein coding RNA 2023	-2.284**	-2.886***
TC14000678.hg.1	MEG8	Maternally expressed 8	-1.982**	-1.028*
TC04000172.hg.1	AC092834.1	unnamed	-1.610***	-1.711**
TC04001939.hg.1	AC092834.1	unnamed	-1.546***	-1.664**
TC01005699.hg.1	AL365361.1	unnamed	-1.318*	-1.558**
TC12002119.hg.1	LINC02441 (CRAT8)	Long intergenic non-protein coding RNA 2441 (cervical cancer-associated transcript 8)	-1.315**	-1.166*
TC21000464.hg.1	B3GALT5-AS1	B3GALT5 antisense RNA 1	-1.304*	-1.171*
TC12003163.hg.1	LINC02441 (CRAT8)	Long intergenic non-protein coding RNA 2441 (cervical cancer-associated transcript 8)	-1.273**	-1.099**
TC03001242.hg.1	THRB-IT1	THRB intronic transcript 1	-1.150**	-1.326***
TC01005698.hg.1	AL365361.1	unnamed	-1.122**	-1.240**
TC06003749.hg.1	LINC02535	Long intergenic non-protein coding RNA 2535	-1.066*	-1.192*
TC16000386.hg.1	AC140658.1 / AC142086.4	unnamed	-1.061*	-1.553*
TC02002653.hg.1	AC019330.1	unnamed	-1.056**	-1.379***
TC01001358.hg.1	LINC01133	Long intergenic non-protein coding RNA 1133	-1.000**	-1.033*
TC08001627.hg.1	CCAT1 / CASC19	Colon cancer associated transcript 1	3.260***	2.837**
TC08001626.hg.1	CASC19	Cancer susceptibility 19	2.002***	1.607*
TC05000500.hg.1	LINC02163	Long intergenic non-protein coding RNA 2163	1.460*	1.014*
TC03000170.hg.1	AC123023.1	unnamed	1.145*	1.803*
TC07001045.hg.1	AC021218.1	unnamed	1.127**	1.086**
Overlapping lncRNAs in the CRC vs. N & CRC vs. Ad comparisons				
HTA 2.0 ID	Annotation	lncRNA name	log2FC (Ad vs. N)	log2FC (CRC vs. N)
TC20000085.hg.1	LINC01752	Long intergenic non-protein coding RNA 1752	-1.117**	-1.091*
TC19002012.hg.1	UCA1	Urothelial cancer associated 1	1.167*	2.048*
TC19000279.hg.1	UCA1	Urothelial cancer associated 1	1.080*	1.976*
TC08000748.hg.1	PCAT1	Prostate cancer associated transcript 1	1.006*	1.251*

*p < 0.05; **p < 0.01; ***p < 0.001

The group of transcripts with altered expression only in Ad vs. N comparison contained 2 downregulated (XIST, PP7080) and 9 upregulated lncRNAs (top 5 with the highest logFC: AC124067.4, LINC01594, DLGAP1-AS2, LINC00261. AL365226.2) (Fig. 2a, Additional file 1: Figure S1/A, Additional file 2: Table S1). Further along the adenoma-carcinoma sequence, lncRNAs with altered expression between CRC and adenoma samples can play a role in the transition of dysplasia-carcinoma that contained 8 downregulated lncRNAs (top 5 with the lowest log2FC AL133370.1, LINC00261, AC005833.1, LINC01612, LINC01594) (Fig. 2a, Additional file 1: Figure S1/B, Additional file 2: Table S1). Among the lncRNAs showing significant expression alteration exclusively in the CRC vs. N comparison, 12 downregulated (top 5 with the lowest log2FC: SNHG14, MIR3936HG, AC124312.3, AL928768.1, AL359397.1) and 5 significantly upregulated lncRNAs (AL645939.4, AC126365.1, LINC00152, AC016831.1, LINC02474) were detected (Fig. 2a, Additional file 1: Figure S1/C, Additional file 2: Table S1).

### Absolute quantification

**Fig. 3:**
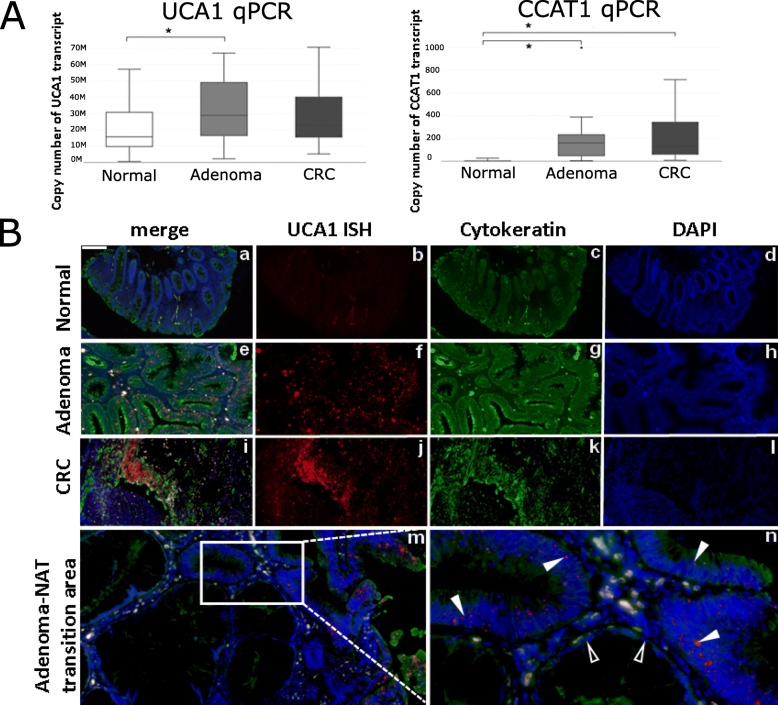
qRT-PCR and ISH analyses of selected lncRNAs. **A**) UCA1 and CCAT1 qRT-PCR absolute quantification on healthy normal (*n* = 20), adenoma (n = 20) and CRC (n = 20) biopsy samples *p < 0.05: UCA1: Ad vs. N, CCAT1: Ad vs. N, CRC vs. N; **B**) Representative UCA1 *in situ* hybridization results on normal (a-d), adenoma (e-h) and CRC (i-l) FFPE tissue samples. UCA1 expression is seen already in adenoma (full arrowhead) compared to monolayered normal adjacent (NAT) mucosa with no ISH signal (empty arrowhead) (m, n) and UCA1 is focally abundant in CRC (j). White signals represent autofluorescence in blood cells and vessels. Digital microscope slides were scanned with Pannoramic 250 Flash instrument (v1.11.25.0), and analyzed with a Pannoramic Viewer (v1.11.43.0) samples, scale bar: 100 μm (a-m) and 40 μm (n)

By the use of absolute quantification, we were able to confirm the lncRNA expression alterations observed by HTA 2.0 analyses. CCAT1 and UCA1 showed upregulation in tumor samples compared to normal tissues (Fig. 3a). In the case of LINC00261, we observed upregulation in adenomas compared to normal controls and downregulation in CRC samples compared to adenomas, but these expression alterations were not significant (data not shown).

### *In situ* hybridization

In normal colonic FFPE tissue samples, no UCA1 ISH signal was detected, whereas UCA1 ISH signal was observed in adenoma tissue and an even stronger signal was detected in colorectal carcinoma samples, in accordance with our qRT-PCR results. The UCA1 ISH signal was localized predominantly in the epithelial cells in adenoma and carcinoma tissue samples (Fig. 3b).

### *In silico* validation on an independent HTA 2.0 dataset

**Fig. 4:**
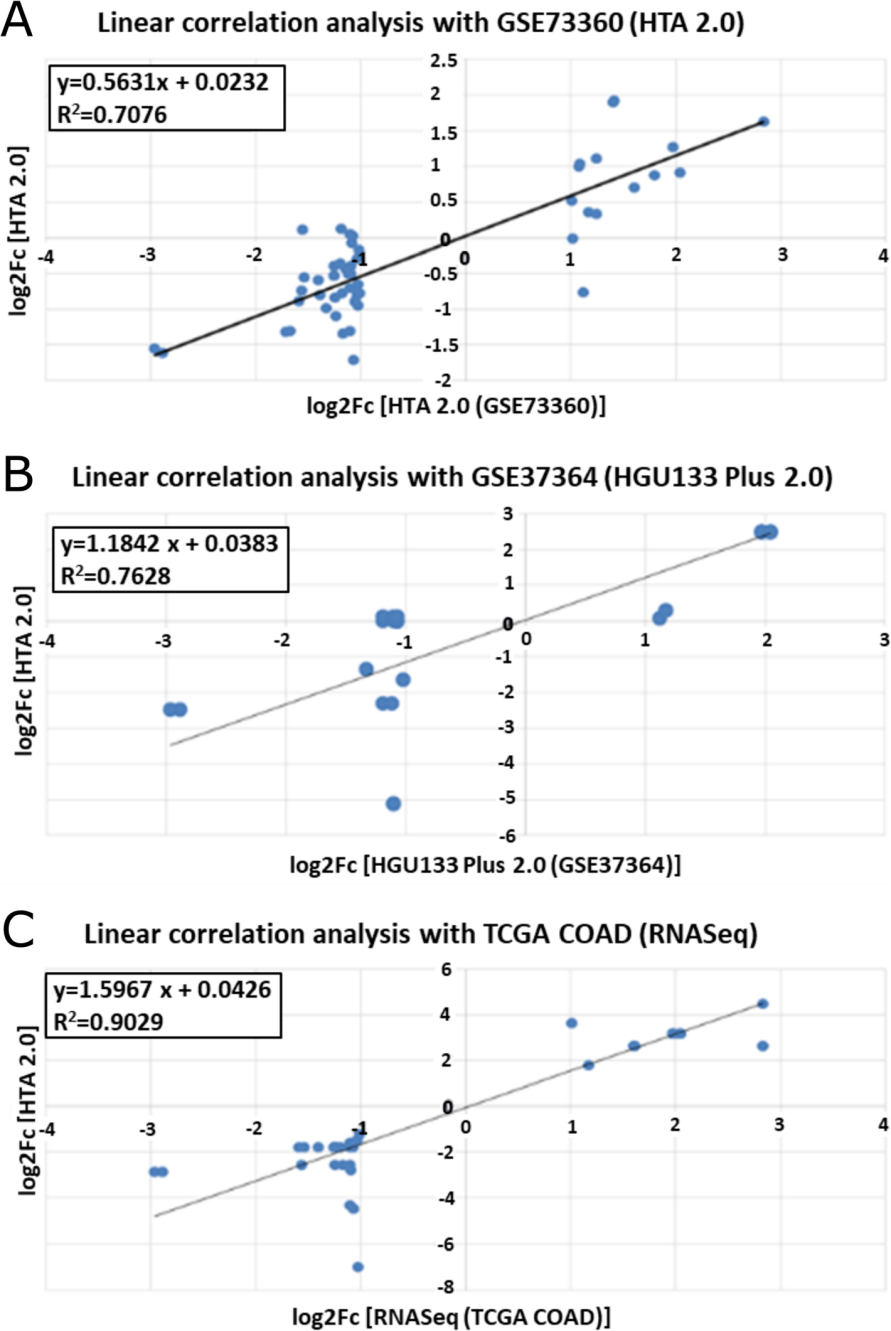
*In silico* validation of HTA 2.0 data on independent datasets. **A**) Linear correlation with an independent HTA 2.0 GEO dataset (GSE73360) [16] [LINC02023, AC092834.1, SNHG14, AL365361.1, AC140658.1, AC142086.4, AC019330.1, THRB-IT1, LINC02535, B3GALT5-AS1, LINC02441, MIR3936HG, AC124312.3, AL928768.1, AL359397.2, LINC01752, AL355922.2, AC087379.1, AC011891.1, AC044802.2, LINC01133, MEG8, CDKN2B-AS1, AC025423.4, LINC01268, CCAT1, UCA1, AC123023.1, CASC19, AL645939.4, AC126365.1, LINC00152, AC016831.1, AC021218.1, LINC02474, PCAT1, LINC02163], **B**) Linear correlation with an independent HGU133 Plus 2.0 dataset (GSE37364) [17] [UCA1, LINC00152, AC016831.1 LINC02023, THRB-IT1, LINC02535, SNHG14, AC124312.3, AL928768.1, CDKN2B-AS1] **C**) Linear correlation with TCGA COAD dataset [18] [LINC01133, AL365361.1, LINC00152, LINC02023, LINC02163, LINC01268, CASC19, CDKN2B-AS1, AC087379.1, LINC02441, SNHG14, AC124312.3, UCA1, LINC01752, CCAT1, AL928768.1]. Our own HTA 2.0 data (GSE100179) are represented on X-axis

Our HTA 2.0 results were compared with an independent HTA 2.0 dataset [17]. The significantly altered lncRNA set in the CRC vs. normal comparison showed the same tendency, a high positive correlation was found with the independent GSE73360 dataset in the CRC vs. NAT comparison (R^2^ = 0.7076) (Fig. 4a, Additional file 3: Table S2).

### *In silico* validation on an independent HGU133Plus2.0 microarray dataset

In order to assess the observed altered lncRNA expression using independent hybridization-based results, HTA 2.0 results were compared for lncRNAs available on the HGU133Plus2.0 microarray platform (GSE37364) [18]. According to the above-mentioned analysis, the expression alterations of 3 significantly upregulated (UCA1, LINC00152, AC016831.1) and 7 downregulated (LINC02023, THRB-IT1, LINC02535, SNHG14, AC124312.3, AL928768.1, CDKN2B-AS1) lncRNAs were verified in CRCs compared to the healthy controls (*p* < 0.01). The expression differences between the HTA 2.0 and HGU133Plus2.0 platforms showed a high positive correlation (R^2^ = 0.7628) (Fig. 4b, Additional file 3: Table S2).

### *In silico* validation on TCGA COAD RNASeq dataset

Out of the 37 lncRNAs identified in our cohort showing significantly differential expression in the CRC vs. N comparison, 16 were detected on the TCGA COAD dataset [19]. A very high positive correlation (R^2^ = 0.9029) was observed between the datasets (Fig. 4c, Additional file 3: Table S2).

### Co-expression analysis of differentially expressed lncRNAs-mRNAs-miRNAs

**Fig. 5:**
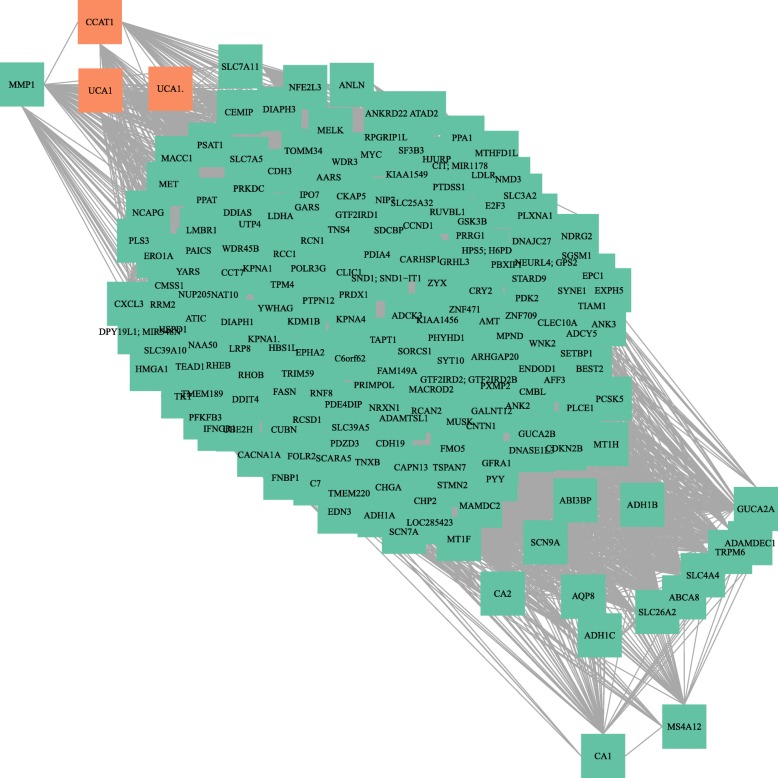
Co-expression network of lncRNAs with associated mRNAs. qPCR validated lncRNAs (UCA1 [TC19000279.hg.1, TC19002012.hg.1], CCAT1 [TC08001627.hg.1]) are represented with orange rectangles, target mRNAs are represented with green rectangles in the network, where further associations between the mRNA targets are also represented. The network was visualized with igraph in R environment and Gene Ontology (GO) functional analysis was performed using TAC 4.0 software (Affymetrix)

mRNAs negatively correlated with CCAT1 are involved in G1/S transition of mitotic cell cycle, G2/M transition of mitotic cell cycle functions, while the positively correlated mRNAs play a role e.g. in cell division and cell cycle regulation. The negatively co-expressed mRNAs with UCA1 have a negative regulatory role of transcription from RNA polymerase II promoter, angiogenesis, DNA methylation, while the positively correlated mRNAs are involved in mitotic cytokinesis and apoptotic processes. The co-expression network of lncRNAs and mRNAs showed certain overlap between the mRNA targets of the selected lncRNAs (Fig. 5 , Additional file 4: Table S3).

**Fig. 6:**
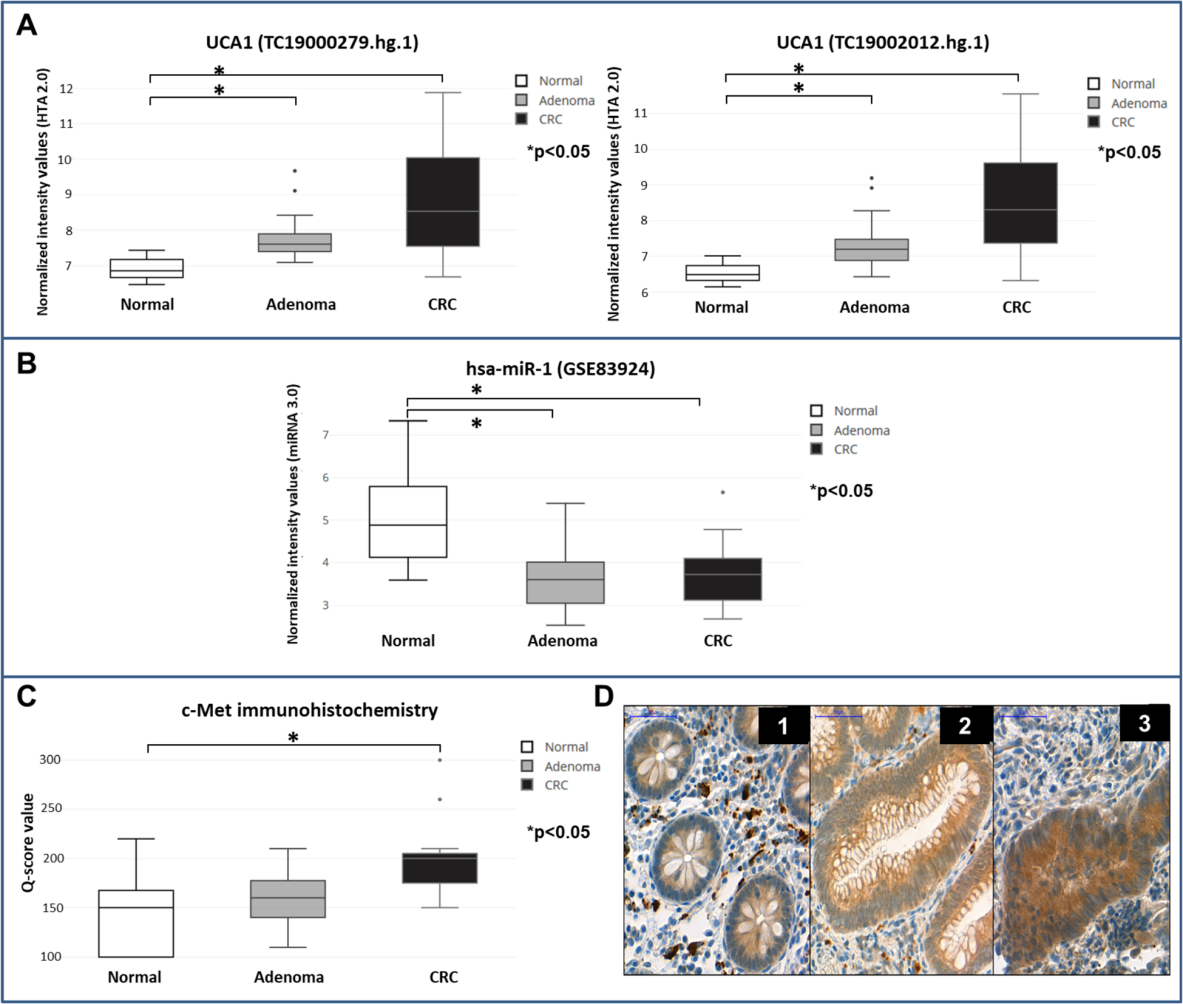
**A**) UCA-1 lncRNA expression in healthy colon, colorectal adenoma, and CRC tissue samples; **B**) hsa-miR-1 expression on GSE83924 miRNA 3.0 array dataset [20] **C**) C-Met expression quantification and **D**) C-Met protein levels in the epithelial compartments of normal colon (D/1), adenoma (D/2) and CRC samples (D/3). Digital microscope slides were scanned with Pannoramic 250 Flash instrument (v1.11.25.0), and analyzed with a Pannoramic Viewer (v1.11.43.0), 40x magnification, scale bar: 50 μm) Box plots represent the median and standard deviation of the data (*p < 0.05)

UCA1 was upregulated in adenoma and also in CRC samples (Fig. 6a) and a potential interaction was predicted between UCA1 and hsa-miR-1 based on independent algorithms. In the GSE83924 dataset [21] hsa-miR-1 was downregulated in adenoma and CRC samples compared to normal colonic samples (*p* < 0.01) (Fig. 6b). MIRWALK validated target prediction revealed that c-Met mRNA is one of the targets of hsa-miR-1. On the other hand, co-expression analysis showed that c-Met was among the most positively co-expressed mRNAs with both IDs of UCA1 (TC19002012: rho = 0.7811; TC19000279.hg.1: rho = 0.7717).

### Immunohistochemistry

In normal epithelium the c-Met expression was mild, diffuse and cytoplasmic (main scoring value: + 1; Q-score: 144.00 ± 49.29). Compared to normal samples, adenoma samples showed elevated, but not significantly increased (*p* = 0.31) epithelial protein expression (scoring values: + 1 and + 2, Q-score: 160.00 ± 29.51). In the epithelial compartment of CRC cases, heterogeneous, moderate/strong (scoring values: + 2 and + 3, Q-score: 201.66 ± 43.02) c-Met protein expression was observed which was significantly higher than in healthy controls (*p* < 0.05) (Fig. 6c, d).

## Discussion

The present study aimed to identify differentially expressed lncRNAs at the whole genome level characteristic of colorectal adenoma and carcinoma tissue samples principally focusing on the adenoma-carcinoma transition. Besides the detection of single lncRNA alterations (by PCR-based methods, Northern blot, RNA-immunoprecipitation and *in situ* hybridization [27]), genome-wide analysis can be achieved by RNA-Seq [28] and also by microarrays, e.g. by Human Transcriptome Array 2.0, a high-resolution microarray platform detecting lncRNA, miRNA and mRNA levels in parallel from the same sample [29].

Certain lncRNAs were previously analyzed in colorectal diseases, but the focus of most of the studies was limited to one or a small set of lncRNAs [30–32]. The strength of our study was the inclusion of colorectal adenoma tissue samples along with the CRC cases in a genome-wide lncRNA expression analysis, in order to identify markers for the early detection of colorectal tumors. Based on the present study, 54 lncRNAs were found to be differentially expressed along the colorectal adenoma-carcinoma sequence that could be validated on independent microarray results and also on TCGA COAD dataset. A subset of lncRNAs already associated with CRC development showed remarkable expression changes in the precancerous colonic adenoma lesions.

The lncRNAs that play a key role during the malignant transformation in CRC development remain to be identified, and therefore, we further focused on the colorectal-adenoma transition. According to our microarray analysis, 12 lncRNAs were downregulated and 5 were upregulated both in adenoma and CRC samples compared to the healthy controls. These lncRNAs might have a key regulatory role during CRC formation, as their dysregulation could be detected already in adenomas, that persisted until the malignant transition. Among these possible key factors, CASC19 overexpression was reported in CRC tissue samples and it was associated with metastasis formation [33]; its knockdown resulted in reduced migration of RKO and Caco2 cells [33]. Upregulation of LINC02163 was also observed in CRC tissue samples by NGS technology [34]. In rectal adenocarcinoma cases Zhang et al. found an association between LINC02163 expression and overall survival [35]. LINC02163 overexpression was documented in gastric cancer tissues and cell lines; knockdown of LINC02163 resulted in reduced cell growth and invasion [36]. Upregulation of AC021218.2 (CRCAL-1) was detected in 3 pairs of CRC compared to their matched normal mucosa by RNA sequencing [37]. According to the current literature in this subset, AL365226.2, LINC02023, AC092834.1, LINC02441, B3GALT5-AS1, THRB-IT1, LINC02535, AC140658.1, and AC142086 have not been reported to be associated with CRC formation and these may serve as novel lncRNA markers of neoplastic processes of the colon.

As in the case of certain presumably ‘driver’ lncRNAs (e.g. MEG8) in the present study, a distinct expression tendency could be detected in adenoma and carcinoma samples, so that their dysregulation may not be a gradual event during cancer formation. It is well-known that up- or downregulation of lncRNAs can be restricted to a certain stage during cancer formation and therefore it is not evident that all differentially expressed RNAs show the same expression tendency in adenoma and carcinoma samples [21]. It is known, that lncRNAs show cell-specific expression in certain tumors [38] and the overall resultant expression found in biopsy samples can be influenced by the epithelial-stromal cell ratio which may differ between colorectal adenomas and adenocarcinomas. On the other hand, similarly to the findings on miRNA [21] and on DNA methylation levels [39], certain lncRNAs showed distinct expression alterations in adenomas and carcinomas that might be due to the counteracting cell-proliferation control pathways in adenomas that are dysregulated in carcinomas.

LncRNAs characteristic only in the malignant state could be identified in the intersection of CRC vs. N and CRC vs. Ad comparisons, where significant downregulation of LINC01752 and overexpression of PCAT1 and UCA1 were observed. Although the altered expression of these lncRNAs are hypothetically associated with malignancy in colonic tumors, due to the relatively low number of CRCs analyzed in the present study, no reliable further conclusion can be formed about the correlation of their expression levels with survival, metastasis or tumor stage data in our cohort. However, on the basis of literature data, overexpression of PCAT1 was suggested to be an independent prognostic factor for CRC [40] and its downregulation inhibited proliferation, blocked cell cycle transition, suppressed cyclin and c-myc expression and induced apoptosis [41]. Silencing of PCAT1 in Caco− 2 and HT-29 cell lines suppressed cell motility, invasiveness, sensitized the cells to 5-FU [42]. Human urothelial carcinoma associated 1 (UCA1) lncRNA has a role in cell proliferation, apoptosis, and cell cycle progression regulation and an increased UCA1 expression level was reported first in urothelial cancer [43] and also reported for breast cancer and CRC patients [44–46]. According to Ni et al., CRC patients with high UCA1 expression had a poor prognosis and UCA1 overexpression was found to be an independent predictor of CRC [47]. Exogenous expression of UCA1 enhanced tumorigenicity, invasive potential, and drug resistance in human bladder TCC BLS-211 cells [43]. Silencing of UCA1 in HCT116, HT29, SW480 and LoVo cell lines significantly decreased cell proliferation, while UCA1 overexpression promoted tumorigenicity in LoVo cells [48]. Silencing of UCA1 suppressed proliferation and metastasis and induced apoptosis of oral squamous cell carcinoma cell lines, which may be related to the activation of the WNT/β-catenin signaling pathway [49]. In a recent study, UCA1 expression was also detected in liquid biopsy samples, where Tao et al. reported that the elevated expression level in the tissue samples can also be detected in plasma samples of CRC patients [50]. The above-mentioned functions of UCA1 illustrate the complexity of its mechanism of actions and its diverse role in CRC development [51].

To date, there has been no data reported about UCA1 expression in colorectal adenoma patients. According to our results, UCA1 shows a gradual upregulation in the normal-adenoma-cancer sequence, thus besides its well-established malignancy-associated functions, it holds the possibility to be utilized as an early detection marker for precancerous lesions of the colon, that may contribute to reduce CRC-related deaths in the future.

Interestingly, a slight discrepancy was observed in UCA1 expression between microarray and qRT-PCR methods. By the two probe sets on HTA 2.0 microarray representing UCA1, all 36 transcripts can be detected, while in contrast, qRT-PCR primers used in the present study detect 4 transcripts (namely UCA1-213, UCA1-207, UCA1-201, and UCA1-228). The above-mentioned reason probably contribute to the discrepant expression results observed between the two platforms. Nevertheless, *in situ* hybridization probes could detect all UCA1 transcripts, and as an added advantage, this approach also provide information regarding lncRNA subcellular localization in colonic tissue samples. Tripathi et al. recently reported moderate UCA1 expression using ISH on colorectal cancer tissue [52]. To the best of our knowledge, our study is the first to report evidence of the upregulation of UCA1 lncRNA in colorectal adenomas and cancers predominantly observed in the epithelial cells adding additional evidence for UCA1 overexpression along colorectal cancer formation.

UCA1 has been reported to regulate miRNAs, such as miR-216b and hsa-miR-1 as a miRNA sponge and as a ceRNA of miR-204-5p among others, influencing growth promotion, apoptosis inhibition and 5-FU resistance [48, 53, 54]. The mutual inhibitory effect and the inverse expression of UCA1 and hsa-miR-1 have already been proven in bladder cancer and one functional interaction site was experimentally confirmed between hsa-miR-1 and UCA1 [54]. Furthermore, after the transfection of pre-miR-1 or following the treatment of UCA1 shRNA, cell proliferation and motility decreased in bladder cancer cell lines in an AGO2-mediated manner [54]. Downregulation of miR-1 in tumors compared to the corresponding normal tissue samples is associated with worse prognosis and its lower expression has been reported to reprogram cancer metabolism via the regulation of tumor glycolysis in colorectal cancer [55]. The interaction between hsa-miR-1 and HGFR (c-Met) is well-known in CRC [56]. On the basis of the comprehensive microarray analysis of Nagy et al. [21] and our present results, the downregulation of hsa-miR − 1 along the adenoma-carcinoma sequence was accompanied by c-MET overexpression. Upregulated UCA1 might exert its effect on c-Met protein and therefore can be associated with metastasis formation and CRC progression. Continuous overexpression of c-Met protein was observed along with the development of CRC [57, 58], which could be confirmed in our cohort, as well. On the basis of our co-expression analysis, c-Met was among the most positively co-expressed mRNAs of UCA1. Taken together, the association of UCA1, hsa-miR-1 and c-Met was predicted by independent algorithms in our study. However, further functional experimental verification is needed to prove the hypothetized interactions in CRC.

Besides the studies aiming at the identification of a signature marker group of lncRNA candidates differentially expressed between CRC and normal samples either on the basis of tissue or plasma samples (e.g. circulating XLOC_006844, LOC152578 and XLOC_000303) [59] or between high-risk and low-risk CRC patients with different overall survival [60], our study identified a subset of lncRNAs potentially playing a key role during the adenoma-carcinoma transition complementing the present knowledge about CRC formation.

## Conclusion

In summary, the defined lncRNA sets may have a regulatory role in the adenoma-carcinoma transition. A subset of lncRNAs showed significant differential expression already in precancerous samples that persisted until CRC formation, raising the possibility of developing adenoma-specific markers in order to achieve early detection of colonic lesions.

## Supplementary information

**Additional file 1: Figure S1.** Differentially expressed lncRNAs in the adenoma-carcinoma sequence. A) Adenoma vs. Normal samples, B) CRC vs. Adenoma samples, C) CRC vs. Normal samples. Intensity values on the color scale are as follows: red – high intensity, black – intermediate intensity, green – low intensity.

**Additional file 2: Table S1.** Differentially expressed lncRNAs in the adenoma-carcinoma sequence.

**Additional file 3: Table S2.** *In silico* validation on independent datasets.

**Additional file 4 : Table S3.** lncRNA-mRNA co-expression network data, GO analysis.

**Additional file 5: Figure S2.** Specific UCA1 *in situ* hybridization signals using RNAScope probes (red stain) on CRC tissue with merged and single channel captures from ISH of UCA1 (Urothelial cancer associated 1), dapB (a Bacillus subtilis gene, negative control probe), and PPIB (Cyclophilin B, positive control probe). Tissue sections were counter stained with DAPI. Digital microscope samples, scale bar: 100 μm.

## Data Availability

The dataset used and analyzed in the present study was uploaded to the Gene Expression Omnibus (GEO) data repository: GEO ID GSE100179 (https://www.ncbi.nlm.nih.gov/geo/query/acc.cgi?acc=GSE100179).

## Abbreviations

5-FU: 5-Fluorouracil
B3GALT5-AS1: B3GALT5 Antisense RNA 1
BACE2-IT1: Beta-secretase intronic transcript 1
CASC19: Cancer susceptibility 19
CCAT1: Colon Cancer Associated Transcript 1
CDKN2B-AS1: CDKN2B Antisense RNA 1
CRC: Colorectal cancer
cRNA: Complementary RNA
CRNDE: Colorectal neoplasia differentially expressed
DLGAP1-AS2: DLGAP1 Antisense RNA 2
GEO: Gene Expression Omnibus
HTA 2.0: Human Transcriptome Array 2.0
ISH: *In situ* hybridization
lncRNA: Long non-coding RNA
MEG3: Maternally expressed 3
MEG8: Maternally expressed 8
MIR3936HG: MIR3936 Host Gene
PCAT1: Prostate cancer associated transcript 1
PVT1: Plasmacytoma variant translocation 1
qPCR: Quantitative polymerase chain reaction
rpm: Round per minute
SNHG14: Small nucleolar RNA host gene 14
sscDNA: Single-stranded complementary DNA
TAC: Transcriptome Analysis Console
TCGA COAD: The Cancer Genome Atlas database, Colon Adenocarcinoma dataset
THRB-IT1: Thyroid hormone receptor beta-intronic transcript 1
TNM: Tumor, node and metastasis staging
TRIS-EDTA: Tris(Hydroxymethyl)aminomethane-ethylenediamine tetraacetic acid
UCA1: Urothelial cancer associated transcript
XACT: X Active specific transcript
XIST: X-Inactive specific transcript
