# Supplementary figures and images for: Genome-wide expression profiling in colorectal cancer focusing on lncRNAs in the adenoma-carcinoma transition
Source: BMC Cancer. 2019 Nov 6;19:1059. doi: 10.1186/s12885-019-6180-5 (PMC6836529; doi:10.1186/s12885-019-6180-5)

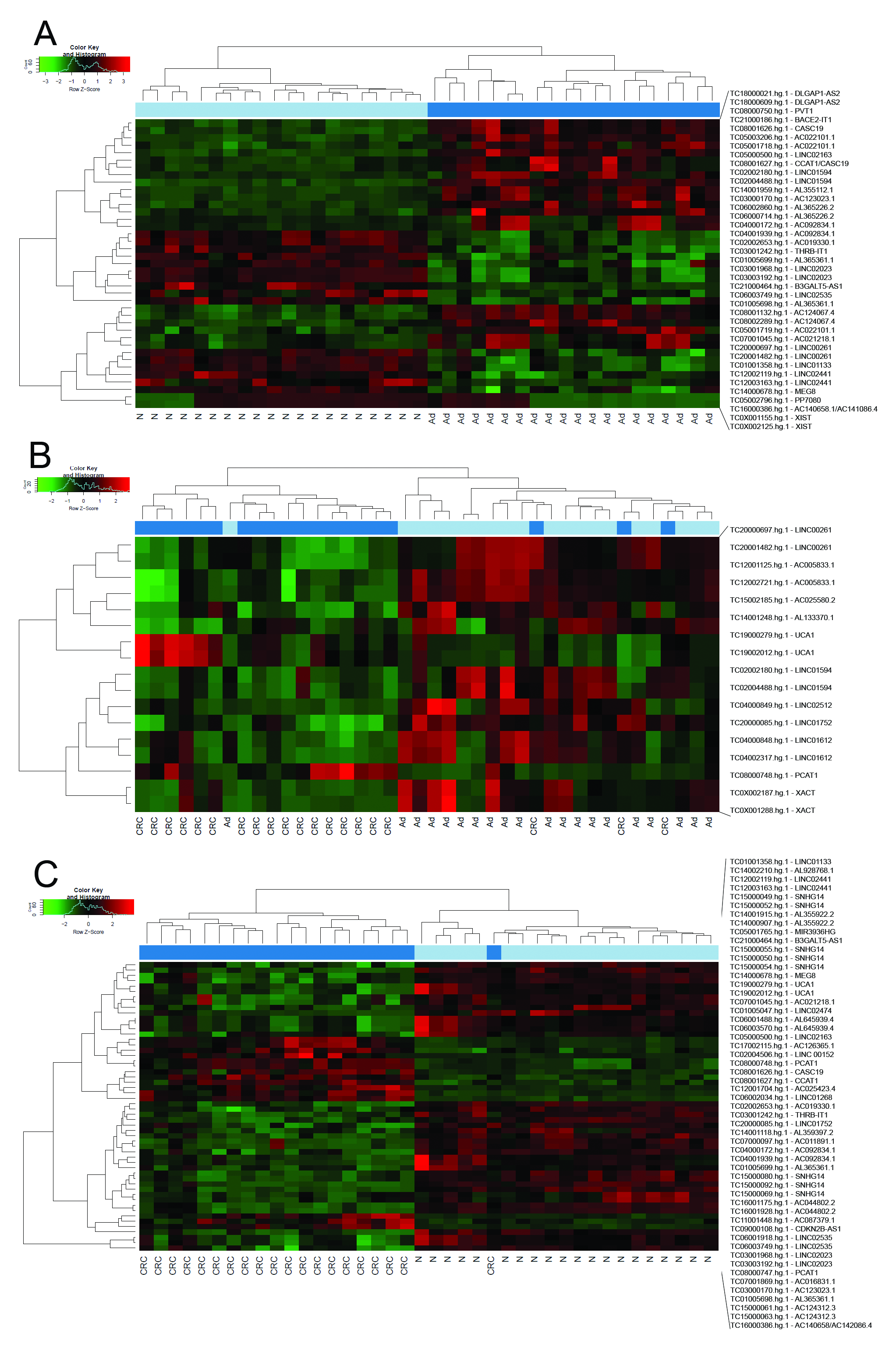

Supplement: Supplementary file 1 — Additional file 1: Figure S1. Differentially expressed lncRNAs in the adenoma-carcinoma sequence. A) Adenoma vs. Normal samples, B) CRC vs. Adenoma samples, C) CRC vs. Normal samples. Intensity values on the color scale are as follows: red – high intensity, black – intermediate intensity, green – low intensity. [file 12885_2019_6180_MOESM1_ESM.tif]

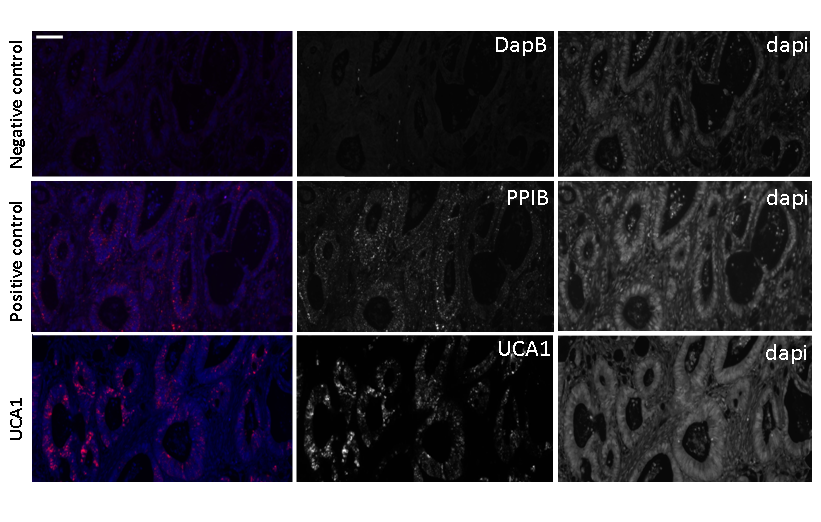

Supplement: Supplementary file 5 — Additional file 5: Figure S2. Specific UCA1 in situ hybridization signals using RNAScope probes (red stain) on CRC tissue with merged and single channel captures from ISH of UCA1 (Urothelial cancer associated 1), dapB (a Bacillus subtilis gene, negative control probe), and PPIB (Cyclophilin B, positive control probe). Tissue sections were counter stained with DAPI. Digital microscope samples, scale bar: 100 μm. [file 12885_2019_6180_MOESM5_ESM.tif]
